# Supplementary material for: Patterning the insect eye: From stochastic to deterministic mechanisms
Source: PLoS Comput Biol. 2018 Nov 15;14(11):e1006363. doi: 10.1371/journal.pcbi.1006363 (PMC6264902; doi:10.1371/journal.pcbi.1006363)
Supplement: S1 Text — (PDF) [file pcbi.1006363.s001.pdf]

Patterning of the Doli eye.

The propagation of errors is described by an additive probability term l_{ij} for each site, (cf. Equation 3) in the main text. This term is defined as

$$l_{i,j+1} = \epsilon \sum_{h \in N(\lfloor j/2 \rfloor)} \left[a_{h,j} - \Theta(p_j - p_{j+1})\right] \exp\left(-\frac{(i-h)^2}{k}\right) \tag{1}$$

with the step function

$$\Theta(x) = \begin{cases} 1 & x \geq 0 \\ 0 & \text{otherwise} \end{cases} . \tag{2}$$

Assuming a stripe pattern, the Θ-term in Eq. (1) gives the a-value expected to be dominant in column j. For $p_j > p_{j+1}$, the probability of green ommatidia ($a_{i,j} = 1$) in column j is larger than that in the adjacent column j. Green ommatidia are the standard in this column j, and a red ommatidium $a_{i,j} = 0$ in a row i is considered an error. Likewise, green ommatidia are considered errors in red-dominated columns ($p_j < p_{j+1}$), where preceding the description holds analogously by exchanging 0 and 1 entries of a.

An error in column j potentially perturbs the nearby ommatidia in the following column $j+1$. The influence is assumed to decay exponentially with the distance between ommatidia; hence the exponential factor with a length scale parameter k in Eq. (1). Influences from all errors in the previous column sum up linearly (summation with row index h) and are scaled with a coupling strength ϵ.

Accounting for the hexagonal lattice, we distinguish two index sets for the row index. The set

$$N(0) = \{1, 2, \ldots, n\} \tag{3}$$

is the usual set of integer index values, used for the columns j with j even. In columns with odd j, sites have row indices in

$$N(1) = \{1.5, 2.5, 3.5, \ldots, n - 0.5\} . \tag{4}$$

Analysis of correlations in patterns.

Given a pattern (a_{ij}) with row index $i \in \{1, \ldots, n\}$ and column index $j \in \{1, \ldots, m\}$, the horizontal autocorrelation coefficient is defined as

$$R^{\mathrm{h}} = \sigma^{-2} n^{-1} (m-1)^{-1} \sum_{i=1}^{n} \sum_{j=1}^{m-1} (a_{ij} - \mu)(a_{i,(j+1)} - \mu) \tag{5}$$

with the pattern's mean value

Fig S1. Partially ordered eyes of flies in the genus Chrysosoma (Dolichopodidae). (A)-(C) and (D)-(F) show two individual eyes that have either high or low measured values for α. Images segmentation was used to identify and classify ommatidial types (shown in (C) and (F)) in order to quantify distributions of ommatidial types.

$$\mu = (nm)^{-1} \sum_{i=1}^{n} \sum_{j=1}^{m} a_{ij} \tag{6}$$

and variance

$$\sigma = -\mu^2 + (nm)^{-1} \sum_{i=1}^{n} \sum_{j=1}^{m} a_{ij}^2 \ . \tag{7}$$

Likewise, the vertical autocorrelation coefficient is defined as

$$R^{\mathrm{v}} = \sigma^{-2}(n-1)^{-1}m^{-1} \sum_{i=1}^{n-1} \sum_{j=1}^{m} (a_{ij} - \mu)(a_{(i+1),j} - \mu) \ . \tag{8}$$

Since we are studying stochastic pattern generation, r realizations ($r \gg 1$) are performed for a given set of parameter values (α, β, P_0). For the pattern generated in each realization k ($1 \leq k \leq r$), the autocorrelation coefficients R_k^{h} and R_k^{v} are computed. Then these coefficients are averaged

$$\langle R^{\mathrm{h}} \rangle = r^{-1} \sum_{k=1}^{r} R_k^{\mathrm{h}} \ , \quad \langle R^{\mathrm{v}} \rangle = r^{-1} \sum_{k=1}^{r} R_k^{\mathrm{v}} \ . \tag{9}$$

Image and data processing

Images of thirty retinas from fifteen individuals (both left and right eyes) were collected using a Leica M80 stereomicroscope and IC80 HD camera. Positions of (x, y) of individual ommatidia were identified using a difference of gaussian blob detection approach as used to classify cell nuclei by Bothma et al. [1]. For each eye, the spatial coordinates of ommatida were scaled so the nearest neighbor of an ommatidium is at distance 1.0 on average. The ratio of red vs. green intensity was used to classify ommatidial type. This way we obtained, for each eye, a list of R ommatidia, $(x_i, y_i, c_i)_{i=1}^{R}$. The i-th ommatidium is characterized by its spatial coordinates x_i and y_i and its type $t_i \in \{\mathrm{red}, \mathrm{green}\}$.

Fig. S1 shows images of two example of eyes from the semi-ordered Chrysosoma species; one is more ordered than the other (high vs. low values for alpha). Real images of eyes are shown on the left, with examples of rotated and zoomed “classified” images on the right. This classified red/green set was used to generate coordinates for each unit eye and assign unit eye type.

The assignment of a column index c_i to each ommatidium i is done as follows.

1. Initially, all column indices are unassigned, $c_i = \varnothing$.
2. The ommatidium closest to the center of mass of all ommatidia is assigned column 0.

3. Among all pairs $P = \{(i,j)|c_j \neq \varnothing, c_i = \varnothing\}$ (of one ommatidium with column assigned, the other unassigned), find the pair (i,j) with smallest Euclidian distance between (x_i, y_i) and (x_j, y_j).
4. If $|y_i - y_j| > |x_i - x_j|$, assign $c_i \leftarrow c_j$. Otherwise: if $x_i < x_j$, assign $x_i \leftarrow x_j - 1$, else $x_i \leftarrow x_j + 1$.
5. If there is an ommatidium with column unassigned, continue at step 3. Otherwise terminate.

This is a heuristic based on the assumption that neighboring ommatidia are in the same column if they are vertically arranged, i.e. the angle between the straight line connecting the ommatidia and the x-axis is larger than $\pi/4$ (45 degrees). We have checked by visual inspection that the heuristic yields a plausible division into columns for the data sets used.

References

1. Bothma J, Magliocco J, Levine M (2011) The Snail repressor inhibits release, not elongation, of paused Pol II in the Drosophila embryo. Curr. Bio. 2011(18):1571–1577.
